# Investigation of the psychometric properties of the German System Thinking Scale in an interprofessional learning setting using the game “Friday Night at the Emergency Room^®^”: a cross-sectional study

**DOI:** 10.1186/s12909-025-07564-2

**Published:** 2025-07-01

**Authors:** Teresa Schmahl, Christoph Strumann, Katja Goetz, Jost Steinhäuser

**Affiliations:** Institute of Family Medicine, University Medical Center Schleswig-Holstein, Luebeck, Germany

**Keywords:** Medical education, System thinking, Serious games, System Thinking Scale

## Abstract

**Background:**

System thinking is recommended as a necessary component in health education. However, it is not yet regularly addressed in medicine and health care professions. Therefore, the simulation game “Friday Night at the Emergency Room^®^” was used to teach system thinking in an interprofessional setting. The aims of this study were to evaluate (a) the psychometric properties of the translated German version of the System Thinking Scale in an interprofessional workshop and (b) the effect of the game on the students’ attitudes toward system thinking.

**Methods:**

The translation of the game and the System Thinking Scale involved independent forward translation, the creation of a consensus version in an interdisciplinary team, cultural adaptation, and a backward translation. Descriptive item analysis and confirmatory factor analysis, internal consistency and reliability of the German System Thinking Scale were calculated. The Interprofessional Socialization and Valuing Scale as well as the evaluation of the effect of the simulation game “Friday Night at the Emergency Room^®^” on the German System Thinking Scale were used for external validation.

**Results:**

The translation of the game and the System Thinking Scale was successful without major cultural adaptations. A total of 97 students (response rate: 37%) from medicine, applied nursing sciences, midwifery, health and health care sciences, physical therapy and occupational therapy, on average 23.3 years old and 67 females (72.8%) participated in the workshop and the overall evaluation. The German System Thinking Scale showed a high internal consistency (Cronbach’s = 0.876) and high level of acceptance. The game had a positive impact on the students’ system thinking, as nine of the 20 items increased significantly after the game.

**Conclusions:**

The German version of the System Thinking Scale is a reliable instrument in measuring system thinking of medical and health care students and it revealed good to moderate psychometric properties. The measured system thinking could be significantly improved by using the game “Friday Night at the Emergency Room^®^” in educational programs.

**Supplementary Information:**

The online version contains supplementary material available at 10.1186/s12909-025-07564-2.

## Background

System thinking is considered a key component of quality improvement, a required attitude to provide safe patient care and a critical core competence for all health care professionals [1]. System thinking is defined as the ability to understand processes, predict system behavior, improve system design, and identify and synthesize patterns, interactions, and dependencies in a set of activities designed for a specific purpose [2–4]. The term has emerged in health care due to the numerous complex, interdependent systems involved, and the recognition that the application of system thinking plays a crucial role in ensuring safe and high-quality care [2]. It is an approach that helps to address patient needs in a systematic process to ensure the best care is delivered [5, 6]. System thinking can lead to better staff performance and improved creativity, integrates practices and promotes public health [7, 8]. Demonstrating skills in system thinking is important for healthcare professionals to effectively engage in successful quality improvement [9].

Years of fixed structures and looking at care from only one perspective at a time led to silo thinking, which can become a barrier to innovation and health system changes [10]. Silo thinking is characterized by not knowing what others are doing, being stuck, isolation, powerlessness and a lack of trust, respect, collegiality and cooperation. This results in the creation of barriers to communication and in the development of disjointed work processes with negative consequences to the organization, employees and patients [11]. System thinking enables to overcome monodisciplinary “silo thinking” and to initiate interprofessionality early. Thereby, it also supports collaborative and non-hierarchical relationships in effective teams [12–14].

Therefore, system thinking is recommended as a useful component in health education and should already be addressed there [12, 15]. The system thinking approach can help students develop important skills by identifying patterns, understanding the causes of problems, analyzing contributing factors and designing systematic improvement processes [1]. For instance, in nursing, the integration of system thinking has been identified as a key of effective leadership, quality and safety improvement, and contemporary competency [16–18]. So far, system thinking has been used in education to improve patient safety and to identify care errors [1, 19–22]. For example, one study showed that teaching system thinking to nursing students and medical students improved the reporting of adverse events [20]. Additionally, nurses with higher levels of system thinking reported higher safety culture and less medication errors [23, 24], higher levels of safety attitude, knowledge, and skill [25]. The results show also that system thinking can change safety monitoring behaviour. Suitable methods for teaching system thinking are courses such as quality improvement, interprofessional education (IPE), error mitigation, and advocacy [1].

However, the concept of system thinking has not yet been consistently integrated into curricula of health care professionals [2, 4, 21, 26] and students rarely receive formal training regarding system thinking during didactic components of their professional training [27]. As a consequence, medical and health care students are not adequately prepared for these management tasks [26, 28]. However, it should be possible to introduce system thinking in undergraduate medical education in the context of patient care. As patient safety is predominantly related to system errors [29], system thinking in health care should be embedded in students’ curricula [21, 30]. Not only it is challenging to develop ways to teach these skills, but it is also challenging to measure the ability to think dynamically and systemically [31]. Therefore, the System Thinking Scale (STS) is an instrument with evidence of its reliability and validity in context healthcare and aims to evaluate the effectiveness of educational efforts to improve system thinking [32].

Evidence-based teaching is important, so instruments with evidence of its reliability and validity are needed to measure the success of teaching in system thinking in Germany. However, there is currently no such scale for measuring system thinking in the German-speaking educational context. Furthermore, the existing system thinking scale had to be adapted to the German healthcare system due to its fragmentation and complexity.

One promising way to teach system thinking is through simulation games. Simulation games (or game-based learning, educational games, serious games) are interactive teaching methods for teaching purposes in the education or training of health professions [29, 33, 34]. These are increasingly being used in medical education [35]. A review shows that there is conflicting evidence on in support of competition in game-based education as an effective teaching strategy for medical students [36]. However, it can be assumed that the help of simulation games, learning should take place in a competitive and active environment that promotes critical thinking, problem solving, collaboration and communication, as well as activating cognitive learning processes [34, 37]. Compared to traditional teaching methods, simulation of real-world scenes links theory to practice in a controlled learning environment [38]. They can be effective tools for providing students with basics for successful interprofessional work during their pre-professional preparation [39].

A simulation game that has already been used successfully in educational institutions internationally, is the board game “Friday Night at the Emergency Room^®^” (FNER), which is based on the theoretical foundations of system thinking. It is used to teach skills such as communication, decision making, problem solving and system thinking. The simulation activity further enables learners to uncover implicit biases that may present barriers in changing mental models [21]. In addition, players should gain insight into the effectiveness of their own management skills [40].

As system thinking is important for a high quality and safe patient care, and due to the lack of instruments with evidence of its reliability and validity for measuring system thinking in German healthcare education context, the aims of this study were to evaluate the psychometric properties of the translated German version of the System Thinking Scale (GSTS) and measure the effect of the board game FNER on the students’ attitudes toward system thinking.

## Methods

### Study design

A cross-sectional design was used to evaluate the psychometric properties of the GSTS by administering an online questionnaire to all participating students of the interprofessional day at the University of Luebeck, Germany. This campus-wide event involves second-semester students from various health-related degree programs and includes structured, interprofessional workshops. Through interactive learning experiences, it aims to promote mutual understanding among the health professions and to strengthen team-building competencies at an early stage of academic and professional training. To estimate the effect of the simulation game - one of the interprofessional workshops conducted during the interprofessional day - on the GSTS, a pre-post design was employed using a paper-based version of the GSTS questionnaire.

### Description of the System Thinking Scale

The 20-item STS, developed by Dolansky et al. [32] measures the system thinking construct of system interdependencies in the context of quality improvement. Their psychometric analysis demonstrated a single factor tool with a Cronbach α value of 0.89 and test-retest reliability of 0.74 [32].

### Translation and cultural adaptation of the System Thinking Scale

Before the STS could be used, it had to be translated and culturally adapted. Translation and cultural adaptation followed the Principles of Good Practice for the Translation and Cultural Adaptation Process by the International Society for Pharmacoeconomics and Outcomes Research (ISPOR) task force [41]. Permission was granted from the first author of the original publication describing the development and validation of the STS [32] on March 8, 2024. After obtaining permission, two researchers (CS health economist and TS health care science researcher) independently translated the English versions into German. The results were discussed during two consensus meetings in an interdisciplinary team of one physician (JS), and the two researchers involved in the translation process in order to reconcile the forward translations into a single consensus forward translation. These steps were followed by cognitive interviews, which was done using the think-aloud method [42]. This was conducted with medical students, a psychologist and general practitioners. They were asked to check for understandability, cultural relevance and interpretation of the translation [41]. The authors (JS, CS, TS) reviewed the cognitive interview responses. The scale did not require any content-related changes. All inconsistencies in the translation were discussed with the original STS author and corrected as advised. The people involved in the translation processes are native German speakers, experienced in this method, and fluent in English. A native speaker working independently translated the German version back into English to check for any translation errors. This translator had not been involved in the forward translation. The back translations with explanatory notes were sent to the original author of the STS for review. No changes needed to be made and finally the GSTS version was approved.

### Game description

The game FNER is a tabletop simulation used to teach and develop system thinking and has been used in a multitude of disciplines both within and outside health care [20, 40, 43]. We therefore used the simulation game FNER for the external validation of the GSTS. Four players engage at a board and each player takes over a department in a simulated hospital (emergency department, surgery, intensive care unit and a regular hospital ward). Each player organizes patient care, transfers, patient flow, staffing needs, emergencies, and documentation for their unit. Teams must manage a hospital during a simulated 24-hour period, providing the highest quality of care with the least amount of resources [40]. Players are exposed to time pressure, limited resources, unexpected scenarios, choices and decisions, and a large number of patients. Multiple boards can be played during a session and at the end the results of the hospitals could be compared.

### Translation and cultural adaptation of the game

The Principles of Good Practice for the Translation and Cultural Adaptation Process of the International Society for Pharmacoeconomics and Outcomes Research (ISPOR) task force have been taken into account in the translation and cultural adaptation of the game [41]. Permission for the translation was obtained from the game’s copyright holders. Initially, the translation was done by an author (CS) and an employee of the Institute of Family Medicine at the University of Luebeck, Germany (CSt). In the first step, the forward translation was carried out independently. The translation was then discussed by consensus with a third independent person (JS). Topics were identified that needed to be culturally adapted for the German context. This was followed by a back translation.

The translation of the game was piloted with two nursing students and three medical students. The feedback was used to optimize the use of the game (structure, explanation of objectives and tasks) as well as its embedding in courses (introduction, handling of questions about the process, reflection). The game’s copyright holders approved the final German version of the game.

### Recruitment and data collection

The data were collected in the context of an interprofessional workshop day at the University of Luebeck, Germany. The special feature of this day is that first-year students from different health science programs (medicine, physical therapy, occupational therapy, applied nursing science, health and health care sciences and midwifery) take part in an interdisciplinary workshop. One of these workshops was conducted by the authors (CS and TS) using the game FNER. Each workshop group was expected to have 20 participants. The workshop groups were divided before the start of the workshop to ensure that they were of equal size. Care was also taken in the composition of the groups to ensure that each study program was equally represented and that inter-professionalism was guaranteed. Within the framework of the interprofessional day, the FNER Workshop took place twice. A total of 40 students could therefore participate. The students who took part in the workshop were informed about the study directly in the workshop. By completing the questionnaire, the students agreed to participate in the study. Ethical approval was given by the ethics committee of the University of Luebeck, Germany (2024 − 250).

All participants who participated in the FNER workshop were asked to complete GSTS at the beginning of the workshop and again in the overall evaluation after the workshop. All other participants of the interprofessional day, who did not take part in the workshop were asked to complete the GSTS in the overall evaluation in an online student’s portal after the day as well. All participants of the interprofessional day were informed about the study by e-mail and reminded once. Printed questionnaires completed by workshop participants were manually entered into SPSS, and the online survey was exported accordingly and merged in one dataset. During data preparation, ambiguous answers were classified as missing values. Furthermore, the final dataset was examined for plausibility.

The overall evaluation includes the GSTS, the German version of the Interprofessional Socialization and Valuing Scale (ISVS) [38, 44, 45] and sociodemographic data. In addition, an open-ended question was used to collect comments from the students. The questionnaire could be completed voluntarily and anonymously. Data collection took place in April 2024.

### Description of the workshop

The workshop was held as part of the interprofessional day with two interprofessional groups, one after the other. One session lasted about two hours. The game was played for about an hour. Six sets of games were available for a maximum of 30 students per session. At the beginning of the session, students were informed about the rules, procedures, and objectives of the game. They were then divided into interprofessional groups of four to a maximum of five. At the end of the time, players wrote down their scores on a provided notepad and added them up. The score was calculated from the sub-areas of quality of care and costs. The team with the lowest use of resources (money, staff) and the highest quality of care, won.

**Fig. 1:**
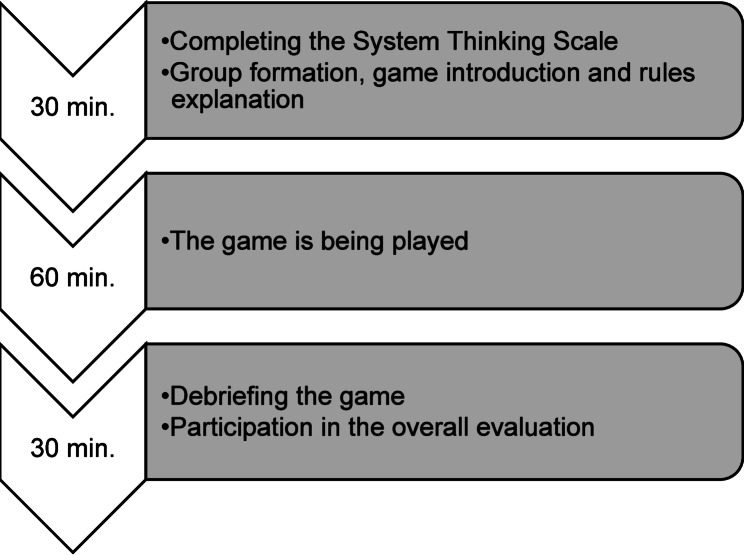
Workshop schedule

This was followed by a debriefing of the game. The students were prompted to engage in reflexive thinking about their actions and decision making, addressing the following questions: “What felt real?”; “What strategies were used during the game?” and “What factors influenced your decision-making?”. Afterwards, the students were familiarized with the theoretical background of the game, i.e., system thinking. Based on the core principles of system thinking (i.e., system parts are interdependent, mental models shape actions, system must adapt to sustain their purpose), students had to develop strategies on how they could have improved their game performance. Finally, based on the students’ practical experience in the health care sector, the reasons why these strategies are not routinely implemented were worked out. The workshop schedule is shown in Fig. 1.

### Statistical analysis

To analyze the psychometric properties, various analyses were performed. Descriptive analysis of sociodemographic data included frequencies with means and standard deviations. Questionnaire responses were analyzed descriptively. Incomplete data points were case-wise deleted. In addition, the data set was examined for outliers. Valid percentages were given, which add up to 100%. In order to obtain an overall evaluation of the GSTS and to test whether the game had an effect on the student’s system thinking, the mean values of all items were calculated and compared with the Wilcoxon sign-rank test before and after the game.

The assessment of psychometric properties included an item response analysis with descriptive statistics for mean, standard deviation, skewness and kurtosis, extent of ceiling and floor effect and corrected item-total correlation. Skewness and kurtosis tests were applied to test for univariate normality [46]. The corrected item-total correlation was calculated to assess whether the individual items measure the same as the other items as a whole. Correlation values less than 0.3 indicate items that do not correlate well with the overall scale [47]. In addition, the acceptability of the items by calculating the proportion of missing or invalid items was assessed. Reliability was described using Cronbach’s alpha as a measure of internal consistency [48], which indicates whether an item on a scale is appropriate for assessing the concept underlying the scale. The values for Cronbach’s alpha range from 0 to 1. The closer the test value is to 0, the less the items are related to each other. Values above 0.60 indicate satisfactory internal consistency and values above 0.80 indicate a high internal consistency. Confirmatory factor analysis (CFA) was used to test the one factor structure of the instrument and to confirm the underlying construct [49]. The Tucker Lewis Index (TLI) and the Comparative Fit Index (CFI) was used to evaluate the goodness of fit of the models. Values that exceed the value of 0.90 can be regarded as an indication of an adequate model fit. Moreover, the Root Mean Square Error of Approximation (RMSEA) was employed to assess the model fit. A value exceeding 0.10 signifies an inadequate fit [49]. The relevance of the individual items was examined by considering standardized factor loadings. Here we consider a magnitude of the standardized factor loadings to be at least above 0.4 as an acceptable range [50]. All items were tested for uni- and multivariate normality. For the latter we applied the Mardia’s multivariate kurtosis and skewness test [51]. In case of rejecting the null hypothesis of multivariate normality, the CFA was estimated with Satorra–Bentler corrected “robust” standard errors. Furthermore, for the fit indices it was used the Satorra-Bentler correction [52]. Due to the limited sample size, we did not estimate CFA for subsets.

To examine the influence of socioeconomic variables on the overall system thinking scores, subgroup analyses were conducted among groups by gender, degree program, and years of experience in health care. Subgroup comparisons were alpproached in an exploratory manner and allowed to explore how different sociodemographic and educational backgrounds influenced system thinking scores.

To assess the relationships with other variables, we compared the outcome of the GSTS scale with the ISVS scale. The ISVS evaluates one’s beliefs, attitudes, and behaviors toward interprofessional collaboration, emphasizing socialization and the value placed on teamwork in healthcare settings. Although, system thinking (GSTS) and interprofessional socialization (ISVS) are distinct constructs they are related because they both have relevance to effective collaborative practice. We therefore expect a correlation, but not a major one.

Further, we compared the means of the GSTS score and of the individual items using the Wilcoxon test before and after the game. As the game was designed to improve participants’ system thinking, we conducted a one-sided hypothesis test, since we expected that the game would enhance students’ system thinking. By focusing on the expected improvement, the one-sided test increases statistical power and reduces the risk of missing a true effect.

The data were analyzed using IBM SPSS Statistics (Version 29.0) and Stata statistical software (Stata version 18, StataCorp, College Station, TX, USA). An alpha level *p* ≤ 0.05 was used for tests of statistical significance.

## Results

### Translation and cultural adaptation

The entire game could be translated without difficulty. There were also few formulations that required cultural adaptation. For example, the term “Step Down” was adapted and translated with the German term “Normalstation”. The step-down department is an intermediate level of care for patients with care needs between the general department and the intensive care unit [53]. In German hospitals, depending on the specialty, patients are usually rather transferred to a regular hospital ward after the intensive care unit [54]. Therefore, this term seemed appropriate in the German-speaking context. Other examples of rewording were: “Ready to Exit” was translated as Transfer/Discharge, “Arrivals” as Patients and “Quality Performance Worksheet” as Worksheet Indicator Set.

The System Thinking Scale has also been minimally adapted to the German language. More information was added to the introduction. The German translated terms for the scale options “Never”, “Seldom”, “Some of the time”, “Often”, “Most of the time” and “Never” were not clear enough to be distinguished from each other. For this reason, we adjusted the rating scale to be linear, with 1 being “most of the time” and 5 being “never”. To interpret the results in an international context all scales were used in reverse order in the German version. The interpretation of the content of a question was discussed with the author and then accordingly translated into German. For the data analysis, the items were rescaled such that a higher score indicates better system thinking. Finally, the translation of the GSTS was presented to the original author. The result of this process is the official German-language version of the game, which is available from the original developers [40].

### Description of the participants

97 students out of a total of 260 students took part in the overall evaluation of the interprofessional day (Response rate: 37.3%), which was used for the psychometric analysis of the GSTS. Students were predominantly female (72.8%) and on average 23.3 years old. The most studied medicine (65.9%). Two third of the students had professional experience in health care. Due to the requirements of the interprofessional day, almost all students were in their first year. Only one student was in the second-year semester as a latecomer.

Of the 260 students, 21 participated in workshop group one and 18 in workshop group two. Of these, 20 students (95.2%) in group one and 18 students in group two (100%) completed the GSTS before and after the game. The same applies to the ISVS in group one, in group two 94.4% completed it. Sociodemographic data were provided by 35 students. Their characteristics were very similar to the group used for the psychometric analysis: 68.6% female, average age 22.6 years and 68.6% were studying medicine. Sample characteristics are shown in Table 1.

**Table 1:** Sample characteristics overall and for the workshop groups

	Psychometric analysis	Workshop Groups
	N = 97*	N = 35*
**Gender, N (%)**		
Female	67 (72.8)	24 (68.6)
Male	21 (27.2)	11 (31.4)
**Age, mean (range**)	23.3 (19–47)	22.6 (20–28)
**Study program, N (%)**		
Applied nursing sciences	8 (8.8)	3 (8.6)
Occupational therapy	4 (4.4)	0
Midwifery	7 (7.7)	3 (8.6)
Medicine	60 (65.9)	24 (68.6)
Health and health care sciences (Master)	5 (5.5)	1 (2.9)
Physical therapy	7 (7.7)	4 (10.3)
**Year of study, y**		
**1**	90 (98.9)	35 (100)
**2**	1 (1.1)	-
**Practical experience in health care**		
**Organizations in years, N (%)**		
0	31/ (34.4)	11 (32.4)
1–4	48 (53.4)	18 (53)
5–7	8 (8.8)	5 (14.7)
> 7	3 (3.3)	

* n varies due to missing data

### Determination of the psychometric properties of the German System Thinking Scale

In relation to the individual items, a maximum of three answers per item were missing (Item 8 to 20). The means of the item’s ratings are between 3.63 and 4.72 (5 for “most of the time” and 1 for “never”). With a mean value of 4.72, Item 3 (I think understanding how the chain of events occurs is crucial) was the item with the highest score. The negative skewness of the items indicates that the distribution of the data was left-tailed, i.e., the majority of observations was above 4. This indicates a rather positive assessment of system thinking. A kurtosis less than zero indicates a broad-peaked distribution.

Corrected item-total correlation ranged from 0.29 to 0.61. The lowest corrected item-total correlation was observed for Item 4: “I include people in my work unit to find a solution” (0.30). The corrected item-total correlation coefficients indicated that all items accounted for a substantial amount of the variance of the total scale and did not differ from each other. The calculated Cronbach’s a of 0.876 showed a high internal consistency. Further details are reported in Table 2.

**Table 2:** Subgroup analysis

Subgroup		*N* (%)	GSTS (SD)	ISVS (SD)	Correlation	*p*-value^a^
**Overall**		97	4.21 (0.40)	5.79 (0.65)	0.31	0.0022
**Female**	Yes	67	4.26 (0.41)	5.79 (0.68)	0.30	0.0182
	No	24	4.05 (0.35)	5.77 (0.62)	0.49	0.0146
	Difference		-0.21	-0.018		
	p-value^b^		0.0400	0.6248		
**Practical experience**	Yes*	31	4.19 (0.42)	5.70 (0.82)	0.27	0.1432
	No	59	4.23 (0.40)	5.90 (0.55)	0.36	0.0058
	Difference		0.04	0.16		
	p-value^b^		0.9934	0.5259		
**Medical** **student**	Yes	31	4.18 (0.40)	5.73 (0.64)	0.18	0.1770
	No	60	4.29 (0.40)	5.90 (0.70)	0.53	0.0033
	Difference		0.10	0.17		
	p-value^b^		0.3314	0.2510		
**Interprofessional experience**	Yes	35	4.31 (0.40)	5.89 (0.58)	0.51	0.0022
	No	21	4.33 (0.48)	5.70 (0.85)	0.29	0.2072
	Difference		0.019	-0.190		
	p-value^b^		0.6116	0.5267		

*No (0) means no practical experience; Yes (1) means ≥ one year of practical experience, SD = Standard Deviation

^a^Pearson Correlation test

^b^Kruskals-Walis Test

Although for some of the items the univariate normality test did not lead to a rejection of the null hypothesis of normality, the multivariate skewness and the kurtosis test lead to a rejection of multivariate normality. Therefore, Satorra–Bentler “robust” standard errors were used in the CFA and Satorra–Bentler scaled and adjusted goodness-of-fit statistics were computed. The estimated CFA fits the data moderately well. While the Satorra–Bentler corrected goodness of fit test (LR = 248.4, *p* < 0.001) is significant indicating that the model does not fit well, the test against independence (LR = 642.1, *p* < 0.001) shows that it is still a significant improvement over a model that assumes the variables are independent. The corrected Bentler’s comparative fit index (CFI = 0.827) as well as the Tucker–Lewis non-normed fit index (TLI = 0.806) do not exceed the value of 0.90 that is usually used to indicate an adequate model fit. The root of mean squared residual was RMSR = 0.0475 and the Satorra-Bentler corrected root mean squared error of approximation was RMSEA = 0.071, 90%-CI= (0.051, 0.089). An inadequate fit is not identified, since the 90%-CI upper bound did not exceed 0.10. All estimated standardized factor loadings were significant, ranging from 0.31 (Item 4) to 0.73 (Item 15). Additional to Item 4, also the items 1, 5 and 12 have standardized factor loadings below the threshold of 0.4. The other items indicated a substantial relevance to the construct of system thinking.

Participants had a tendency toward both ends of the scale per item. Whereby score 1 (“never”) was chosen rather less in comparison. The “ceiling effect” was estimated as 3.1% and a “floor effect” could not be determined (see additional file 1).

### Results of the ISVS

The mean overall score of 5.79 (1 “strongly disagree” and 7 “strongly agree”) on the ISVS suggests that students have a positive perception and attitude toward working with members of other professions. A positive attitude is also evident with regard to the individual items, as none of the mean values of the items are below 5 (see additional file 2).

### Relations with other variables

Table 3 consists of the results of the subgroup analyses as well as the results of the correlation analyses between the GSTS and the ISVS that are used to assess the relationships with other variables. For both the GSTS and the ISVS, differences between gender (female yes/no), practical experience (yes/no), study program (medical student yes/no), and interprofessional experience (yes/no) were examined. Only one variable shows a significant difference between the groups. Females have a significantly higher GSTS score than males (*p* = 0.04). The ISVS score shows no differences among the considered groups.

**Table 3:** Game effect on the GSTS score

Item	Pre-test mean (SD)	Post-test mean (SD)	Mean difference (SD)	*P*-value
1	3.94 (0.70)	4.18 (0.77)	0.24 (0.68)	0.032*
2	4.55 (0.60)	4.44 (0.55)	-0.10 (0.60)	0.113
3	4.60 (0.59)	4.68 (0.52)	0.07 (0.63)	0.307
4	4.52 (0.55)	4.52 (0.60)	0.00 (0.61)	0.500
5	3.92 (0.78)	4.13 (0.74)	0.21 (0.62)	0.038*
6	3.97 (0.68)	4.48 (0.65)	0.51 (0.80)	< 0.001*
7	4.24 (0.68)	4.64 (0.53)	0.40 (0.55)	< 0.001*
8	3.81 (0.83)	4.26 (0.72)	0.44 (0.82)	0.002*
9	3.68 (0.70)	4.18 (0.80)	0.5 (0.76)	< 0.001*
10	4.10 (0.64)	4.39 (0.67)	0.28 (0.83)	0.031*
11	4.28 (0.61)	4.39 (0.71)	0.10 (0.86)	0.270
12	3.89 (0.98)	4.07 (0.88)	0.18 (1.20)	0.188
13	3.52 (0.95)	3.60 (0.94)	0.07 (0.94)	0.395
14	4.44 (0.64)	4.52 (0.72)	0.07 (0.74)	0.333
15	4.10 (0.69)	4.43 (0.68)	0.32 (0.78)	0.014*
16	4.21 (0.57)	4.23 (0.75)	0.02 (0.85)	0.396
17	3.48 (0.73)	4.13 (0.94)	0.64 (1.18)	0.001*
18	4.15 (0.63)	4.26 (0.72)	0.10 (0.79)	0.289
19	3.63 (0.85)	3.84 (1.00)	0.21 (0.87)	0.090
20	4.10 (0.76)	4.36 (0.67)	0.26 (0.68)	0.020*
**Score**	**4.06 (0.31)**	**4.29 (0.43)**	**0.23 (0.28)**	**< 0.001**

^*Significant at the 5%−level; SD = Standard Deviation^

The correlation between the GSTS and the ISVS was weak (0.31), but significant (*p* < 0.002). Regarding the subgroups, differences in the correlation can be detected. For instance, for males there is a higher correlation between GSTS and ISVS (0.49 vs. 0.30). In addition, non-medical students showed a higher correlation and a significant relationship between the GSTS and the ISVS (0.53, *p* = 0.003). This is similar for students who have already gained practical experience (0.51, *p* = 0.002), compared to those who have not (0.29, *p* = 0.207).

### Results of the German System Thinking Scale pre- and posttest

Table 4 shows the means and the standard deviation of each item as well as the score for before and after playing the game based on the workshop participants. The mean of the GSTS before (4.06) and after the game (4.29) show an overall significant increase by 0.23 (*p* < 0.001).

**Table 4:** Item characteristics of the GSTS (*n* = 97)

Item	Missing *N* (%)	Mean	SD	Skewness	Kurtosis	Corrected item-total correlation	Standardized Factor Loadings^c^
1. Seek everyone’s view of the situation	0	4.13	0.70	-0.38	− 0.239	0.30	0.35*
2. Look beyond a specific event to determine the cause of the problem.	1 (1.0)	4.51	0.65	-0.98*	-0.12	0.41	0.47*
3. Understanding how the chain of events occurs is crucial.	1 (1.0)	4.72	0.54	-1.79*	2.35	0.43	0.46*
4. Include people to find a solution.	1 (1.0)	4.59	0.55	-0.95*	-0.11	0.30	0.30*
5. Patterns are more important than any one specific event.	1 (1.0)	4.02	0.86	-0.35	-0.87*	0.32	0.34*
6. Think of the Problem at hand as a series of connected issues.	2 (2.1)	4.17	0.75	-0.60*	-0.04	0.52	0.56*
7. Consider the cause and effect that is occurring in a situation.	1 (1.0)	4.41	0.63	-0.56*	-0.59	0.56	0.66*
8. Consider the relationships among co-workers.	3 (3.1)	4.01	0.81	-0.39	-0.49	0.59	0.57*
9. Systems are constantly changing.	3 (3.1)	3.95	0.80	-0.30	-0.48	0.46	0.48*
10. Propose solutions that affect the work environment.	3 (3.1)	4.20	0.73	-0.50	-0.35	0.58	0.59*
11. Proposed changes can affect the whole system.	3 (3.1)	4.34	0.63	-0.42	-0.65	0.55	0.63*
12. More than one or two people are needed to have success.	3 (3.1)	4.21	0.84	-0.76*	-0.28	0.33	0.31*
13. Keep the mission and purpose of the organization in mind.	3 (3.1)	3.79	0.97	-0.42	-0.44	0.45	0.51*
14. Small changes can produce important results.	3 (3.1)	4.41	0.65	-0.90*	0.90	0.52	0.57*
15. Consider how multiple changes affect each other.	3 (3.1)	4.30	0.73	-0.70*	-0.19	0.62	0.73*
16. How different employees might be affected by the improvement.	3 (3.1)	4.35	0.56	-0.13	-0.73*	0.57	0.62*
17. Strategies that do not rely on people’s memory.	3 (3.1)	3.63	0.89	-0.40	0.34	0.52	0.53*
18. System problems are influenced by past events.	3 (3.1)	4.23	0.81	-1.20*	2.15*	0.59	0.64*
19. Consider the past history and culture of the work unit.	3 (3.1)	4.01	0.82	-0.85*	1.22*	0.55	0.56*
20. Same action can have differrent effects over time, depending on the state of the system.	3 (3.1)	4.26	0.73	-0.78*	0.43	0.43	0.48*
Total Score	3 (3.1)	4.21	0.40	-0.06*^a^	− 0.451*^b^	Cronbach‘s α = 0.876	

^a^Mardia’s multivariate skewness test; ^b^Mardia’s multivariate kurtosis test; ^c^completely standardized factor loadings (i.e., standardized for both items and the latent construct); *significant at the 5%-level

**Fig. 2:**
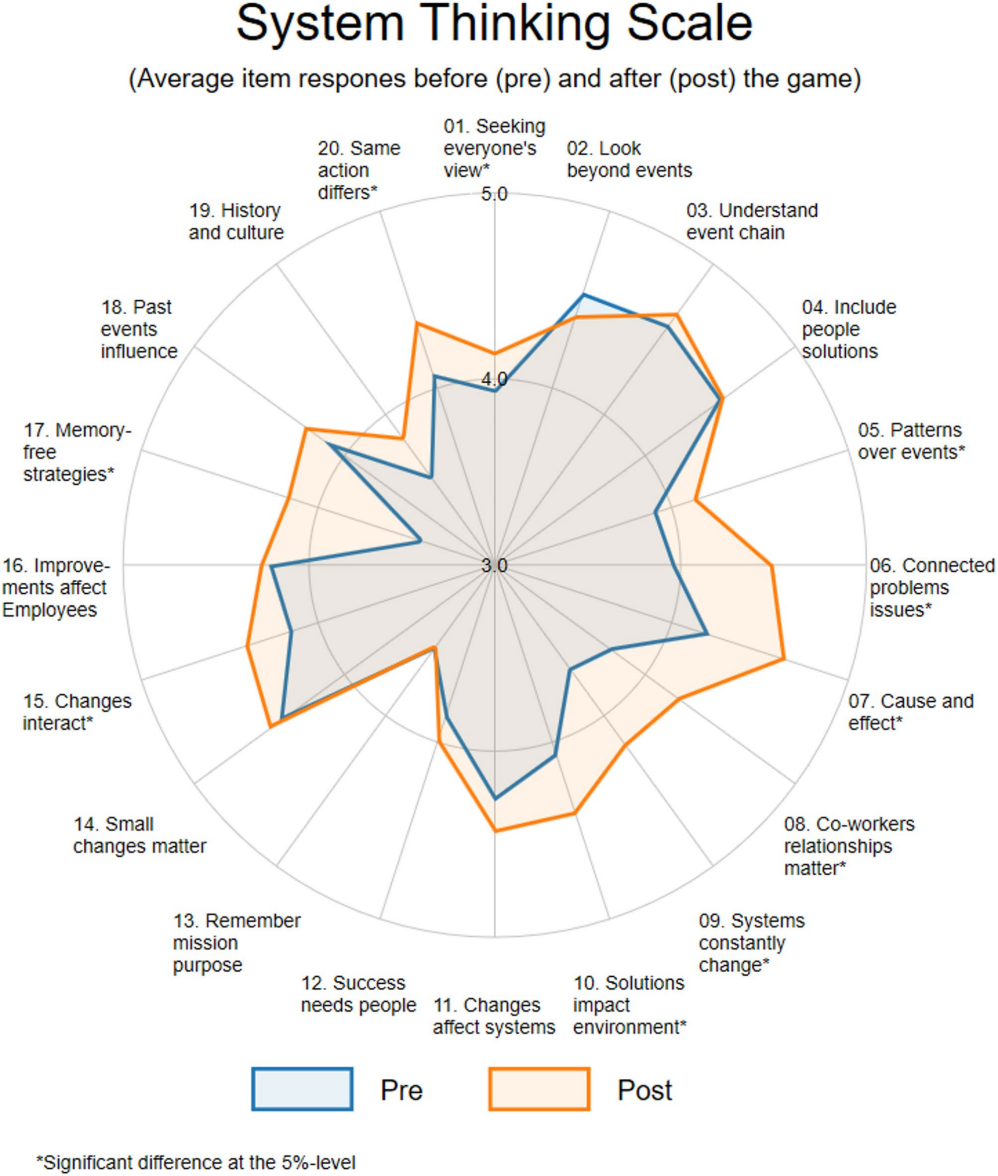
Spider plot

The means of each item of the GSTS for before and after playing the game based on the workshop participants were also shown in a Spider plot provided in Fig. 2. Except for Item 2, the means of all items have increased after playing the game. For nine of the 20-items, significant increases after the game can be observed.

## Discussion

The GSTS as well as the game FNER have been successfully translated into German, culturally adapted and approved. Furthermore, the GSTS was successfully tested for psychometric properties to support its use in an interprofessional educational setting.

The psychometric analysis revealed that the internal consistency of the GSTS is very good with a Cronbach’s alpha of 0.876. This is similar to the original English version (coefficient of 0.89 [32]). The low number of missing answers indicates high acceptance and successful use of the translated GSTS. The fit of the estimated CFA was only moderate and identifies space for improvement. Especially, the items 1 (Seeking everyone’s view), 4 (Include people solutions), 5 (Patterns over events) and 12 (Success needs more than one or two people) have standardized factor loadings below the threshold of 0.4. Future studies should clarify whether the rather weak correlation of these items with the construct of system thinking is due to the student sample lacking work experience or to the nature of the items themselves. The factor loadings of Item 4 and 12 increases above 0.4 if the CFA is estimated based on data of students with working experience. However, future analyses should rely on larger sample sizes.

Due to the significant correlation with the ISVS questionnaire, a good comparability with the translated GSTS can be assumed in our study. Interestingly females had a significantly higher GSTS score than males. Similar differences have not been reported in previous studies. It must also be taken into account that the proportion of women in this study was higher. Therefore, it remains to be investigated whether these differences can be explained by a selection effect or to what extent females have a better understanding of system thinking. Gender differences in cognitive ability are often small, and individual differences and social factors are more important than gender itself [55]. The same can be applied to the results that non-medical students or students with practical experience show a higher correlation and a significant relationship with the GSTS and ISVS scores.

Being able to measure system thinking is important to increase the understanding of the mechanisms by which quality improvement processes achieve their results, assist staff to recognize system contributions to quality improvement, provide valuable information on the effectiveness of interventions, and provide insights into the available human social capital on teams to understand why some teams are more effective than others [32]. Furthermore, on the premise that system thinking can be taught and learnt, the ability to assess system thinking provides a way to measure the effectiveness of educational efforts to improve system thinking.

The game showed a positive impact on perceived system thinking. However, the improvement in score cannot be attributed to the game-play due to a missing control group. Further studies should introduce a control group. Furthermore, as students scored higher on the GSTS after the game than before. However, while the overall score shows a significant increase after playing the game, this holds not for all individual items. Most of the items with insignificant differences before and after the game have means above 4, i.e., the second highest scale of the Likert scale ranging from 1 to 5. Although we did only find weak evidence for a ceiling effect in general (3%), this may limit the potential for increases by the game. For example, Item 3 has a pre-test mean of 4.6 and a post-test mean of 4.68. Instead, the items with insignificant scores have significantly lower pre-test means. The items 12, 13, 16 and 19 also show significant increases, but their pre-test means were below 4. The absence of a significant impact of the game on these items may be attributed to their apparent strong alignment with a professional working context (Item 19: Consider the past history and culture of the work unit) that might not be suitable for students.

A positive influence of the FNER game on system thinking has already been shown in other studies [20, 21, 27]. Sanko and Mckay [20] explored, that people with a higher system thinking level perceive events that are more strongly influenced by systems. Another study investigated the effect of the game in relation to the system thinking scale in an interprofessional setting. As in our study, it was found that the overall scores on the system thinking scale for nursing, pharmacy, dietetics and speech and language pathology students increased significantly from pre-test (M = 82.8, SD = 10.6) to post-test (M = 89.7, SD = 10.8), *p* < 0.001 [27]. Significant pre- and posttest differences in the results of the system thinking scale were also found by Fusco et al. [56] using the game FNER.

Sanko & Mckay [20] were also able to show that the effects of the learned system thinking can be limited in time. It is therefore advisable to include the game and the teaching of system thinking in repeated courses. In this way, learned concepts can be consolidated over a long period of time and skills as well as knowledge can be maintained. The effects of the game and the influence of system thinking on practical activities should be evaluated and compared over a longer period of time. A contextual and process evaluation could examine the implementation, impact mechanisms, and relevant contextual factors of the game and courses in the short, medium, and long term. However, the effects of repeated interventions on individual GSTS items need to be investigated in further studies.

The evaluation of the game was carried out with students in an interprofessional setting. Increased interprofessional collaboration can lead to new perspectives, learning opportunities and outcomes [57]. Interdisciplinary team approaches can reduce systematic errors, health care costs and unwarranted treatments. Therefore, establishing an early and solid foundation for interprofessional collaboration is critical to successful patient outcomes. Interprofessional collaboration in the context of the game FNER improves mutual understanding of roles and perspectives, which leads to comprehensive and real-world learning experiences. The similarity of the results found regardless of the discipline shows that FNER can be used extensively regardless of the discipline. In future, the game can also be used between health-related and non-health-related study programs or across hospital units.

As our and other study results show, system thinking could mitigate medical errors, improve problem-solving and decision-making skills, improve timing and quality of interactions with other professionals and patients [2, 20, 21]. Therefore, teaching system thinking is important because the approach can help students develop foundational skills in recognizing patterns, understanding the root causes of problems, analyzing contributing factors, and designing systematic improvement processes that can withstand the workload and stress of health care professionals [1]. Teaching system thinking is becoming increasingly important for sustainable management in health care because of the increasing complexity, dynamics, and uncertainty of systems and the resulting need for long-term strategic thinking [58]. The teaching requires didactic, experience-oriented and reflective learning. Courses such as quality improvement, interprofessional education, error mitigation, and advocacy as suitable to teach system thinking.

### Limitations

One limitation was that the interprofessional workshop was a one-time event with no control group. Data collection from a single educational setting may limit the ability to generalize the results of this study. However, assuming similar implementation at other institutions, broad applicability is possible. With a response rate of 30% out of a total of 260 students, replication with a larger number of students may provide evidence of the generalizability of the results. In this study the proportion of women is higher than that of men (72.8% female). This can be explained by the usual statistics for these study programs. In the field of human medicine and health sciences, almost three quarters (72%) of first-year students in 2023 in Germany were female [59]. The proportion of women in the participating study programs is high overall. This is also reflected in the overall high proportion of women in the healthcare workforce in Germany (75%) [60]. In addition, the study program of medical students was proportionally the highest compared to the other programs. This is because the study program in medical education at our university is generally larger than the other study programs.

Participation in the game was voluntary, so students who were interested in a game may have been more likely to take part. Therefore, response bias cannot be ruled out. However, the topic of system thinking was not disclosed to the students before the workshop.

Due to the study design, test-retest reliability and responsiveness to change could not be determined and no conclusions about the sensitivity to change can be drawn. The lack of test-retest reliability testing limits insights into the GSTS’s stability over time. Further research should investigate the long-term retention or sustainable effect of the game on system thinking.

In a next step, it would certainly be interesting to find out to what extent socio-cultural (ethnicity) and socio-economic (parents’ socio-economic status) factors of the participants could influence system thinking.

## Conclusions

The GSTS is a reliable instrument with good to moderate psychometric properties. It can fill the existing lack of questionnaires to examine the ability to system thinking in in medical and health education. The game FNER can have a positive effect on students’ system thinking. Given the positive results of this work, further use in teaching students of various health professions and medicine is essential. Our experience indicates the game FNER is a meaningful interprofessional activity that prepares students to practice system thinking in their future careers. In the long term, extensive use of the game and the GSTS in other interprofessional courses is planned. The availability of this instrument encourages further research in this field in German-speaking countries. Future research is needed to continue to assess the psychometrics of the GSTS. The evidence concerning the relations with other variables would be enhanced by testing additional sets of learners at other institutions and/or at different levels of education and capturing qualitative data using structured interviews.

## Electronic Supplementary Material

Below is the link to the electronic supplementary material.

Supplementary Material 1: Additional file 1: Table 1 Item score frequency of the GSTS, n (%)

Supplementary Material 2: Additional file 2: Table 2 Items and total score of the ISVS, *n* = 95

## Data Availability

The dataset supporting the conclusions of this article is included within the article (and its additional file(s)). The German translation of the System Thinking Scale can be requested for use from the authors.
