# Supplementary material for: Investigation of the psychometric properties of the German System Thinking Scale in an interprofessional learning setting using the game “Friday Night at the Emergency Room®”: a cross-sectional study
Source: BMC Med Educ. 2025 Jul 1;25:875. doi: 10.1186/s12909-025-07564-2 (PMC12211719; doi:10.1186/s12909-025-07564-2)
Supplement: Supplementary file 1 — Supplementary Material 1: Additional file 1: Table 1 Item score frequency of the GSTS, n (%) [file 12909_2025_7564_MOESM1_ESM.docx]

Additional file 1

**Table 1** Item score frequency of the DSDS, n (%)

| Item | 1 | 2 | 3 | 4 | 5 |
| --- | --- | --- | --- | --- | --- |
| 1 | - | 51 (52.6) | 15 (15.5) | 1 (1) | 30 (30.9) |
| 2 | - | 31 (32.3) | 8 (8.3) | - | 57 (59.4) |
| 3 | - | 19 (19.8) | 4 (4.2) | - | 73 (76.0) |
| 4 | - | 33 (34.4) | 3 (3.1) | - | 60 (62.5) |
| 5 | - | 35 (36.5) | 25 (26) | 3 (3.1) | 33 (34.4) |
| 6 | - | 45 (47.4) | 14 (14.7) | 2 (2.1) | 34 (35.8) |
| 7 | - | 43 (44.8) | 7 (7.3) | - | 46 (47.9) |
| 8 | - | 42 (44.7) | 21 (22.3) | 3 (3.2) | 28 (29.8) |
| 9 | - | 44 (46.8) | 23 (24.5) | 3 (3.2) | 24 (25.5) |
| 10 | - | 44 (46.8) | 14 (14.9) | 1 (1.1) | 35 (37.2) |
| 11 | - | 46 (48.9) | 8 (8.5) | - | 40 (42.6) |
| 12 | - | 33 (35.1) | 16 (17) | 3 (3.2) | 42 (44.7) |
| 13 | 1 (1.1) | 34 (36.2) | 26 (27.7) | 8 (8.5) | 25 (26.6) |
| 14 | - | 42 (44.7) | 5 (5.3) | 1 (1.1) | 46 (48.9) |
| 15 | - | 39 (41.5) | 12 (12.8) | 1 (1.1) | 42 (44.7) |
| 16 | - | 53 (56.4) | 4 (4.3) | - | 37 (39.4) |
| 17 | 2 (2.1) | 38 (40.4) | 34 (36.2) | 5 (5.3) | 15 (16) |
| 18 | 1 (1.1) | 42 (44.7) | 10 (10.6) | 2 (2.1) | 39 (41.5) |
| 19 | 1 (1.1) | 48 (51.1) | 16 (17) | 3 (3.2) | 26 (27.7) |
| 20 | - | 44 (46.8) | 10 (10.6) | 2 (2.1) | 38 (40.4) |
