# Supplementary material for: Investigation of the psychometric properties of the German System Thinking Scale in an interprofessional learning setting using the game “Friday Night at the Emergency Room®”: a cross-sectional study
Source: BMC Med Educ. 2025 Jul 1;25:875. doi: 10.1186/s12909-025-07564-2 (PMC12211719; doi:10.1186/s12909-025-07564-2)
Supplement: Supplementary file 2 — Supplementary Material 2: Additional file 2: Table 2 Items and total score of the ISVS, n = 95 [file 12909_2025_7564_MOESM2_ESM.docx]

Additional file 2

**Table 2** Items and total score of the ISVS, n = 95

| **Item** | | **Mean** | **SD** |
| --- | --- | --- | --- |
| 1 | Aware of my preconceived ideas | 5.48 | .95 |
| 2 | Using a common language | 6.12 | .79 |
| 3 | Awareness of my own role | 5.56 | 1.02 |
| 4 | Share and exchange ideas | 6.06 | 1.02 |
| 5 | Perception of myself as someone who is involved in interprofessional practice | 5.46 | 1.33 |
| 6 | Feel comfortable being the leader | 5.06 | 1.40 |
| 7 | Feel comfortable in speaking out within the team | 5.55 | 1.31 |
| 8 | Feel comfortable in describing my professional role | 5.83 | .96 |
| 9 | Sharing research results in a team | 6.02 | .98 |
| 10 | Able to negotiate more openly with others | 5.55 | 1.24 |
| 11 | Gained an enhanced awareness of roles of other professionals | 5.72 | .97 |
| 12 | Comfortable engaging in shared decision-making | 5.90 | 1.00 |
| 13 | Feel comfortable in accepting responsibilities delegated to me | 5.93 | .92 |
| 14 | Better understanding of the client’s involvement in decision-making | 5.75 | 1.15 |
| 15 | Feel comfortable clarifying misconceptions with other members | 5.67 | 1.18 |
| 16 | Gained greater appreciation of the importance of a team approach | 6.30 | .71 |
| 17 | Act as a fully collaborative member of the team | 6.25 | .85 |
| 18 | Initiating discussions about sharing responsibility | 5.77 | 1.00 |
| 19 | Sharing decision-making with other professionals | 6.13 | 1.02 |
| 20 | Realistic expectations of other professionals | 5.70 | .95 |
| 21 | Gained an appreciation for the benefits in interprofessional team work | 6.31 | .93 |
| **Overall ISVS-Score** | | **5.79** | **.64** |
